# Comparative Mechanistic Insights and Therapeutic Potential of Pembrolizumab, Durvalumab, and Ipilimumab as Immune Checkpoint Inhibitors in the Targeted Management of Oral and Head and Neck Squamous Cell Carcinoma

**DOI:** 10.3390/cancers17172805

**Published:** 2025-08-27

**Authors:** Piotr Kawczak, Igor Jarosław Feszak, Tomasz Bączek

**Affiliations:** 1 Department of Pharmaceutical Chemistry, Faculty of Pharmacy, Medical University of Gdańsk, 80-416 Gdańsk, Poland; tomasz.baczek@gumed.edu.pl; 2 Institute of Health Sciences, Pomeranian University in Słupsk, 76-200 Słupsk, Poland; igorfeszak@gmail.com; 3 Department of Nursing and Medical Rescue, Institute of Health Sciences, Pomeranian University in Słupsk, 76-200 Słupsk, Poland

**Keywords:** ICI, targeted treatment, PD-1i, PD-L1i, CTLA-4i, OSCC, HNSCC

## Abstract

Immune checkpoint inhibitors (ICIs) have transformed the landscape of cancer therapy by reactivating immune surveillance mechanisms against tumor cells. In the context of oral squamous cell carcinoma (OSCC) and broader head and neck squamous cell carcinoma (HNSCC), agents such as pembrolizumab, durvalumab, and ipilimumab target PD-1, PD-L1, and CTLA-4, respectively. This review comprehensively examines their clinical efficacy, safety profiles, mechanisms of action, and therapeutic potential in OSCC management, with an emphasis on strategies to overcome therapeutic resistance. A systematic analysis of the literature was conducted, focusing on clinical outcomes, ongoing trials, and emerging combination therapies. Pembrolizumab has demonstrated significant improvements in overall survival (OS) and progression-free survival (PFS) in OSCC patients. Durvalumab, mainly utilized in locally advanced or recurrent disease, has shown survival benefit, particularly in combination or maintenance settings. Ipilimumab exhibits durable responses in advanced OSCC, with enhanced efficacy observed when used alongside nivolumab in dual checkpoint blockade regimens. Although both pembrolizumab and nivolumab target PD-1, they differ in clinical indications and regulatory approvals. Notably, ICIs are associated with immune-related adverse events (irAEs), requiring careful monitoring. Collectively, these agents represent promising therapeutic options in oral cancer, though future studies must prioritize the identification of predictive biomarkers and the development of optimized combination strategies to maximize therapeutic benefit while minimizing toxicity.

## Simple Summary

Oral cancer remains a significant clinical challenge, particularly in its advanced stages, where therapeutic options are limited and prognosis is often poor. Recent advancements in immunotherapy, particularly the development of immune checkpoint inhibitors, have demonstrated considerable potential by enhancing the immune system’s ability to recognize and eliminate tumor cells. This review examines three key immune checkpoint inhibitors—pembrolizumab, durvalumab, and ipilimumab—each targeting distinct immune regulatory pathways to potentiate antitumor immunity in oral cancer. The primary objective is to evaluate their therapeutic efficacy and safety profiles and to assess emerging strategies aimed at overcoming treatment resistance. Insights from this analysis may inform clinical decision-making, optimize treatment combinations, and guide future research toward minimizing adverse effects while maximizing clinical benefit.

## 1. Introduction

Oral squamous cell carcinoma (OSCC) is the most common oral malignancy, comprising over 90% of cases and posing a major global health challenge, especially in South and Southeast Asia, where tobacco, alcohol, and betel quid use are prevalent [1,2]. In contrast, HPV-associated squamous cell carcinoma—predominantly HPV-16–driven—is increasing mainly in the oropharynx, particularly in Western populations [3,4]. In 2020, oral cancer accounted for over 377,000 new cases and approximately 177,700 deaths worldwide [1].

OSCC typically affects middle-aged to elderly men, exhibits aggressive behavior, and has a 5-year survival rate of only 50–60%, largely due to late diagnosis and early metastasis [5,6]. Standard treatments—surgery, radiotherapy, and chemotherapy—are effective in early disease but less so in advanced or recurrent cases and often cause significant morbidity [7].

Immune checkpoint inhibitors (ICIs) have redefined cancer therapy by blocking pathways such as PD-1/PD-L1 and CTLA-4, thereby restoring antitumor immunity and altering the tumor microenvironment (TME) [8,9,10]. Systematic reviews and meta-analyses confirm ICIs improve OS and PFS in various cancers [11,12,13]. Additional evidence from meta-analyses further supports these benefits across tumor types [14,15].

In recurrent or metastatic OSCC and other head and HNSCC, PD-1 inhibitors like pembrolizumab and nivolumab provide superior outcomes [16,17]. Other clinical studies reinforce these findings in advanced HNSCC [18]. Increased PD-1/PD-L1 expression in oral potentially malignant disorders (OPMDs) suggests a role for early immunotherapy [19]. Compared with conventional therapies, ICIs offer better efficacy and lower toxicity in advanced HNSCC. EGFR-targeted therapy with cetuximab is also used but is limited by tumor heterogeneity and resistance [20,21].

Groundbreaking immune checkpoint research, awarded the 2018 Nobel Prize, led to agents such as ipilimumab and pembrolizumab, which have shown durable responses in melanoma and HNSCC [22]. CTLA-4 inhibitors (ipilimumab and tremelimumab) act during T-cell priming [23,24,25], while PD-1 inhibitors (cemiplimab, nivolumab, and pembrolizumab) counter tumor-mediated suppression [26,27]. Similarly, PD-L1 inhibitors (atezolizumab, avelumab, and durvalumab) block tumor-driven immune evasion [28,29]. Pembrolizumab (KEYNOTE-048) and nivolumab (CheckMate-141) are FDA-approved for recurrent/metastatic HNSCC, with each occupying distinct clinical niches [30,31,32,33]. Durvalumab shows promise in combination regimens (CONDOR, EAGLE) [34,35], and ipilimumab, although less effective alone in OSCC, may enhance PD-1 inhibitor activity [35,36,37].

Resistance to ICIs—primary, acquired, or adaptive—remains a major hurdle [38,39]. Strategies under investigation include combining ICIs with immunogenic cell death inducers [40]; targeting innate immune checkpoints [41]; and integrating mRNA-based cancer vaccines with anti-PD-1, anti-PD-L1, or anti-CTLA-4 antibodies [42]. Future efforts should focus on next-generation ICIs and optimized combination regimens, guided by a deeper understanding of tumor immunobiology and resistance mechanisms, to further improve outcomes [43,44,45].

Table 1 highlights pembrolizumab, durvalumab, and ipilimumab as key ICIs in cancer immunotherapy, summarizing ongoing trials and associated adverse effects.

Despite the clinical success of ICIs in OSCC and other malignancies, major challenges remain due to heterogeneous patient responses and the absence of reliable predictive biomarkers. Although PD-L1 expression, tumor mutational burden (TMB), and tumor-infiltrating lymphocytes have been explored, their prognostic and predictive value in OSCC remains uncertain [49]. Immune evasion is further promoted by regulatory T cells (Tregs) and myeloid-derived suppressor cells (MDSCs), which suppress effective antitumor immunity [50].

ICI response is shaped by tumor-intrinsic and extrinsic factors beyond PD-L1 expression. The gut microbiota has emerged as a critical modulator, with taxa such as *Akkermansia muciniphila* and *Bifidobacterium* spp. linked to enhanced PD-1 blockade efficacy, while antibiotic-induced dysbiosis reduces treatment benefit [51,52,53]. High TMB correlates with increased neoantigen load, promoting immune recognition and improved survival in multiple cancers, including HNSCC [54,55,56]. Epigenetic alterations, including DNA methylation and histone modification, also facilitate immune escape; targeting these changes may restore immunogenicity and sensitize tumors to ICIs [57,58,59]. The tumor immune microenvironment (TIME) is pivotal—“hot” tumors with abundant CD8^+^ T cells and interferon activity respond better to ICIs than “cold” tumors with limited immune infiltration or dominant suppressive cell populations [60,61,62]. These findings support a multifactorial biomarker approach integrating microbiome composition, TMB, epigenetics, and immune contexture for personalized immunotherapy in OSCC.

Targeting oncogenic signaling offers another strategy. The PI3K/Akt/mTOR pathway promotes tumor proliferation and immune suppression [63,64]. Additionally, this pathway drives PD-L1 upregulation, contributing to ICI resistance [65,66]. Inhibiting this axis restores T-cell infiltration and enhances ICI efficacy in preclinical OSCC models [67,68], though toxicity and variable clinical responses remain limiting factors [69,70,71]. Similarly, aberrant Wnt/β-catenin signaling impairs dendritic cell recruitment, reduces T-cell priming, promotes Treg and MDSC expansion, and fosters immune exclusion, driving resistance to PD-1/PD-L1 blockade [72,73,74,75]. While targeting Wnt/β-catenin in OSCC is challenging due to pathway complexity and off-target toxicity [76,77,78], combined inhibition of PI3K/Akt/mTOR and Wnt/β-catenin holds promise for overcoming resistance [79].

Combining ICIs with chemoradiation is under investigation, but concerns persist regarding toxicity and patient selection [80,81]. While these combinations may enhance immune activation, they also heighten the risk of mucositis, dermatitis, hematologic toxicity, and irAEs such as pneumonitis, colitis, and endocrinopathies [82,83,84]. Optimizing timing and sequencing is essential to balance efficacy and safety [85], and biomarker-driven patient stratification may improve outcomes. Ongoing trials aim to define evidence-based protocols [86,87]. ICIs can also cause multi-organ irAEs [88,89,90]. These include rare but serious cases of ICI-induced diabetes [91,92,93,94] and may exacerbate pre-existing autoimmune diseases [95,96,97]. Antibiotics can impair ICI efficacy [98]. Oral-specific toxicities—such as xerostomia, lichenoid reactions, and candidiasis—are underreported but clinically important [99,100,101]. Additional studies further document these oral toxicities in patients receiving ICIs [102,103,104]. Evidence continues to accumulate across larger cohorts and case series [105,106,107], with further reports reinforcing their clinical impact [108,109,110]. More recent investigations also highlight these toxicities [111,112,113,114]. Collectively, these findings underscore the need for standardized management [115].

This review summarizes the evolving OSCC and HNSCC therapeutic landscape, focusing on ICI mechanisms, clinical outcomes, and challenges, as well as emerging molecular targets such as PI3K/Akt/mTOR and Wnt/β-catenin. The literature was identified through PubMed searches (2015–2025) using terms “pembrolizumab”, “durvalumab”, and “ipilimumab” with “oral cancer”, prioritizing clinically relevant studies. Ongoing advances in tumor immunology and precision oncology will be crucial to optimizing treatment strategies and improving OSCC outcomes.

## 2. Pembrolizumab—Programmed Cell Death Protein 1 (CD279) Blocker

Pembrolizumab is a humanized IgG4 monoclonal antibody developed by Merck & Co. that targets programmed cell death protein 1 (PD-1), a receptor expressed on activated T cells that downregulates immune responses and facilitates tumor immune evasion [116,117]. Both pembrolizumab and nivolumab are monoclonal antibodies targeting PD-1 receptor, thereby preventing PD-1 engagement with its ligands PD-L1 and PD-L2 and restoring T-cell activity. Despite their shared target, these agents differ in certain clinical and pharmacologic aspects. Pembrolizumab is a humanized IgG4κ antibody, whereas nivolumab is a fully human IgG4 antibody [118]. Clinically, pembrolizumab has demonstrated efficacy in recurrent or metastatic head and neck squamous cell carcinoma (HNSCC), particularly in patients with high PD-L1 expression, as highlighted in the KEYNOTE-048 trial, where pembrolizumab alone or in combination with chemotherapy improved OS compared with cetuximab plus chemotherapy [119]. Nivolumab, evaluated in the CheckMate 141 trial, showed survival benefits in platinum-refractory HNSCC, regardless of PD-L1 status. These differences emphasize pembrolizumab’s preferential use in first-line treatment for PD-L1-positive patients, while nivolumab remains a key option in the post-platinum setting. Moreover, dosing schedules differ slightly, with pembrolizumab commonly administered every 3 or 6 weeks, while nivolumab is typically dosed every 2 or 4 weeks [30].

Initially approved by the U.S. Food and Drug Administration (FDA) in 2014 for metastatic melanoma, pembrolizumab marked a pivotal milestone in cancer immunotherapy [120,121,122]. Its indications have since expanded to multiple malignancies—including non-small cell lung cancer, HNSCC, urothelial carcinoma, and oral cancer—driven by significant survival benefits demonstrated in clinical trials [123,124,125].

Structurally, pembrolizumab is derived from a mouse anti-human PD-1 antibody and engineered onto a human IgG4 scaffold incorporating the S228P mutation. This modification stabilizes disulfide bonds, prevents Fab-arm exchange, and enhances antibody specificity. It also contains a glycosylation site at Asn297, with glycans positioned internally to reduce effector function. These design features minimize Fc gamma receptor and C1q complement binding, thereby limiting antibody-dependent cytotoxicity and complement activation. While such refinements aim to reduce immunogenicity and off-target immune activation, irAEs remain possible and may necessitate treatment interruption, underscoring the need for vigilant safety monitoring [126,127,128].

Table 2 summarizes the effector immune responses elicited by PD-1 blockade.

Pembrolizumab is administered intravenously at 1–10 mg/mL over approximately 30 min, demonstrating dose-proportional pharmacokinetics and a terminal half-life of ~26 days [130,131,132]. Steady-state levels are typically achieved by week 18 with triweekly dosing [133,134,135]. Clearance is influenced by body weight but generally unaffected by mild hepatic or renal impairment [136,137,138]. These pharmacological properties have been characterized extensively in the KEYNOTE clinical trial program across multiple malignancies [139,140,141,142,143,144,145,146,147].

Mechanistically, pembrolizumab blocks PD-1 binding to its ligands PD-L1 and PD-L2, a checkpoint axis frequently exploited by tumors to evade immune surveillance [148]. Under physiological conditions, PD-1 signaling suppresses T-cell activity, facilitating tumor progression [149,150]. Inhibiting this interaction restores T-cell function, enabling immune-mediated tumor recognition and destruction, and has led to durable responses and improved survival in diverse cancers [151,152,153,154].

Since its initial approval for metastatic melanoma, pembrolizumab has become integral in oncology [155,156,157]. Its indications now span non-small cell lung cancer, HNSCC, urothelial carcinoma, and other malignancies [158,159,160]. Additional studies further support its use across diverse cancer types [161,162,163,164]. In metastatic melanoma, it outperforms conventional chemotherapy in survival outcomes [165,166]. In NSCLC, it is a first-line option for PD-L1–positive tumors, offering superior progression-free and overall survival [167,168]. For HNSCC, including OSCC, it is particularly effective in recurrent or metastatic disease, especially in PD-L1–expressing tumors [121,169], and is also approved for advanced urothelial carcinoma [170,171].

In OSCC, pembrolizumab has shown promise for advanced disease where standard modalities often fail [172]. Clinical studies report improved outcomes in PD-L1–positive tumors [173,174]. A phase II trial in recurrent or metastatic HNSCC, including OSCC, found an objective response rate of 16.7% [175]. As a second-line option post–platinum chemotherapy, it provides survival benefits [176,177]. However, intratumoral PD-L1 heterogeneity limits predictive accuracy [178]. Ongoing research is focused on novel biomarkers and combination strategies, including chemotherapy and radiotherapy, to overcome resistance and enhance efficacy [179,180,181,182,183].

Pembrolizumab’s safety profile in OSCC mirrors that in other cancers. irAEs—such as dermatitis, colitis, hepatitis, and pneumonitis—are the most frequent, resulting from immune activation [184,185]. These are typically manageable with corticosteroids or dose adjustments, supporting a favorable risk–benefit ratio [186,187].

As a PD-1/PD-L1 axis inhibitor, pembrolizumab has improved outcomes across multiple tumor types, yet challenges remain in patient selection and immune-related adverse event (irAE) management [188,189]. Ongoing trials are evaluating its integration with chemoradiation and its use across disease stages to improve survival and quality of life in OSCC [190,191]. Pembrolizumab is now considered a cornerstone in HNSCC therapy [192,193,194]. However, treatment response varies with PD-L1 expression, tumor mutational burden, and host immunity [195,196,197]. These factors contribute to primary resistance [198,199,200,201,202]. Acquired resistance can also diminish benefit, and real-world outcomes may differ from trial results due to patient comorbidities and performance status [203,204].

Though generally tolerable, pembrolizumab can cause multi-organ irAEs—including thyroiditis, hypophysitis, and pneumonitis—that may require immunosuppression or treatment discontinuation [205,206,207,208]. Patients with pre-existing autoimmune disease face higher complication risks [209,210,211]. The absence of robust predictive biomarkers and standardized selection criteria underscores the need for personalized strategies and improved toxicity management protocols.

**Figure 1:**
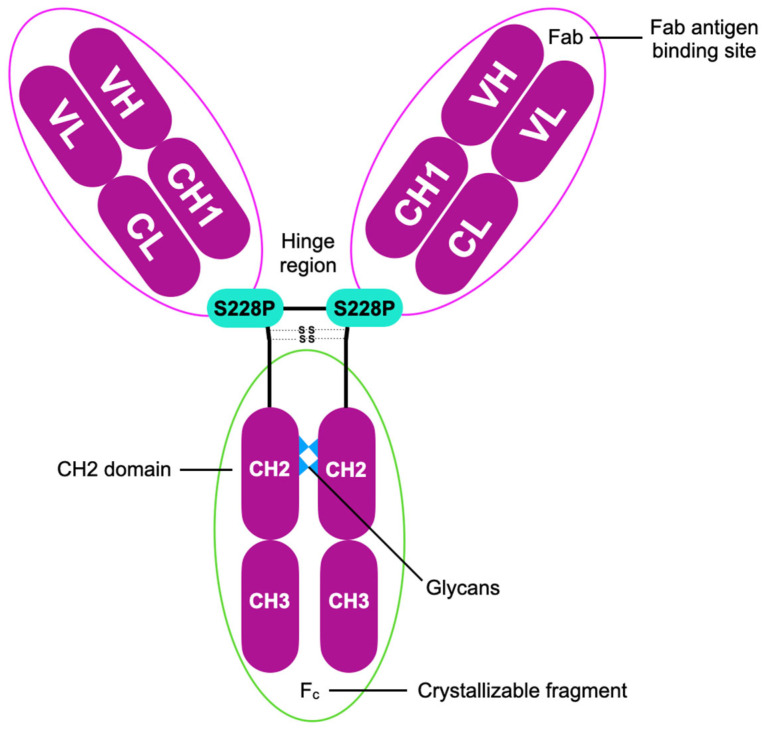
Structural features and essential characteristics of pembrolizumab—humanized monoclonal IgG4-κ isotype antibody containing a stabilizing S228P (Ser228Pro) Fc mutation according to [212]; pembrolizumab consists of constant heavy (CH) and constant light (CL) chains, an antigen-binding fragment (Fab), a crystallizable fragment (Fc), and variable heavy (VH) together with variable light (VL) regions.

Figure 1 displays the molecular structure and key characteristics of pembrolizumab.

## 3. Durvalumab—Programmed Death-Ligand 1 (CD274) Blocker

Durvalumab is a fully human monoclonal antibody developed by AstraZeneca that targets programmed death-ligand 1 (PD-L1), a critical immune checkpoint protein expressed on tumor and antigen-presenting cells [213,214]. By binding PD-L1 and preventing its interaction with PD-1, durvalumab counteracts tumor-induced immune suppression, restoring T-cell activity and enhancing antitumor immune responses [215,216,217]. This mechanism directly disrupts the PD-1/PD-L1 axis—a major pathway exploited by tumors to evade immune surveillance [218,219,220]. In 2017, durvalumab received U.S. Food and Drug Administration (FDA) approval for locally advanced or metastatic urothelial carcinoma after platinum-based chemotherapy, following evidence of improved PFS and OS [221,222,223]. This milestone highlighted the therapeutic relevance of PD-L1 inhibitors and spurred their integration into combination regimens across multiple malignancies [224,225,226,227,228,229,230,231,232,233].

PD-L1 mediates immune escape primarily through PD-1 engagement, which inhibits T-cell activation. Its expression is driven by oncogenic signaling pathways (e.g., AKT, STAT) and genetic alterations, as well as inflammatory stimuli such as interferon-γ. In the TME, immune cells—including dendritic cells and monocytes—also express PD-L1, further contributing to T-cell suppression and regulatory T-cell induction. Beyond immune inhibition, PD-L1 may transmit intracellular signals that increase tumor resistance to cytotoxic T-cell-mediated killing [126].

Head and neck squamous cell carcinoma (HNSCC) is a heterogeneous disease arising from the oral cavity, oropharynx, larynx, and hypopharynx. While tobacco and alcohol remain the predominant risk factors, a subset—especially oropharyngeal cancers of the tonsils and base of tongue—is associated with human papillomavirus (HPV) and exhibits distinct immunologic features. HNSCC typically presents with an immunosuppressive microenvironment characterized by dysregulated immune cell composition and high immune checkpoint expression. PD-1/PD-L1 blockade with agents such as nivolumab, pembrolizumab, durvalumab, and atezolizumab has shown efficacy in select patient subsets, and ongoing trials are exploring their use alongside chemotherapy, radiotherapy, and other immunomodulators, including CTLA-4 and IDO-1 inhibitors [234,235,236].

Durvalumab’s therapeutic effect stems from PD-L1 blockade, which restores T-cell function and promotes tumor destruction, yielding durable responses across cancer types [237,238]. While mechanistically similar to PD-1 inhibitors, its direct PD-L1 targeting may confer advantages in tumors where PD-L1 expression is the primary immune escape mechanism [239,240,241]. After approval in urothelial carcinoma, durvalumab was further indicated for non-small cell lung cancer (NSCLC), particularly as consolidation therapy for patients with locally advanced disease remaining progression-free after chemoradiation. In this setting, durvalumab significantly improves PFS and OS, cementing its role as a standard maintenance therapy [242,243,244,245]. Combination regimens with chemotherapy have expanded durvalumab’s antitumor potential in multiple malignancies [246,247]. Ongoing studies are investigating its role in head and neck, cervical, and hepatocellular carcinomas [248,249,250], with promising preliminary results [251,252,253].

In OSCC, durvalumab is emerging as a viable treatment option, particularly in recurrent or metastatic disease. OSCC often presents at advanced stages with high recurrence rates, making effective systemic therapy crucial. PD-L1 overexpression is common in OSCC, providing a strong rationale for PD-L1 blockade. Early clinical trials in advanced HNSCC, including oral cavity tumors, have demonstrated tumor regression and survival improvement with durvalumab-based regimens [254,255]. PD-L1 has also been proposed as a prognostic biomarker, with evidence suggesting that HGF/Met signaling can upregulate PD-L1, reinforcing its role in immune evasion [256]. Durvalumab is currently under evaluation as a second-line therapy for patients who fail surgery, radiotherapy, or chemotherapy, offering a potential alternative in refractory OSCC [257,258].

The clinical impact of durvalumab in OSCC is linked to disruption of PD-1/PD-L1-mediated immune suppression, a recognized driver of OSCC pathogenesis. High PD-L1 expression correlates with poorer prognosis, supporting the rationale for PD-L1-targeted therapy [259,260]. A multicenter trial showed that durvalumab combined with chemotherapy improved survival and response rates in advanced HNSCC, underscoring the potential value of combination regimens [261]. Such strategies may be particularly useful in OSCC to overcome resistance mechanisms [262].

However, therapeutic response to durvalumab varies, largely due to differences in PD-L1 expression. This underscores the need for biomarker-guided patient selection [263]. Immune-related adverse events remain a concern, with toxicities affecting the skin, gastrointestinal tract, liver, nervous system, muscles, eyes, and salivary glands. Reported events include dermatitis, colitis, hepatitis, encephalitis, myopathy, limbal stem cell deficiency, corneal perforation, IgG4-related pleural disease, hyposalivation, Sjögren’s syndrome, and immune thrombocytopenia. Notably, some irAEs have occurred in patients receiving ICIs after mRNA COVID-19 vaccination [264,265,266]. Others have also been reported in this context [265,266,267], with additional cases described in recent studies [268,269,270,271]. While often manageable with immunosuppression, severe irAEs may require treatment interruption or discontinuation [272,273].

Research efforts now focus on enhancing durvalumab’s efficacy through combination strategies with chemotherapy, radiotherapy, cytokine-based therapies, and other checkpoint inhibitors [274,275,276]. Preliminary clinical data support these approaches in advanced OSCC [254,277]. As understanding of the OSCC immune microenvironment deepens, integrating PD-L1 status and other biomarkers into treatment decision-making is expected to refine patient selection and optimize outcomes [278,279].

Durvalumab remains a cornerstone of modern immuno-oncology. Its blockade of the PD-L1/PD-1 axis has delivered clear clinical benefit in multiple malignancies, including OSCC. Yet, challenges persist—such as heterogeneous PD-L1 expression, irAE management, and the absence of validated predictive biomarkers. These issues limit its precision in clinical practice but also drive ongoing research to maximize survival and quality-of-life benefits in advanced disease. In NSCLC, durvalumab’s greatest impact is seen in unresectable stage III disease following chemoradiotherapy; however, its efficacy diminishes in patients with low or absent PD-L1 expression [246,280]. Real-world outcomes may also be lower than in trials due to older age, comorbidities, and reduced performance status [281,282]. Treatment timing is critical, as delays between chemoradiotherapy and durvalumab initiation correlate with worse outcomes [84]. Safety concerns—particularly pneumonitis, colitis, hepatitis, and endocrine disorders such as thyroid dysfunction and adrenal insufficiency—necessitate vigilant monitoring, especially in patients with prior thoracic radiation or pre-existing autoimmune or pulmonary disease [283,284,285,286].

Regulatory influences of the TME on PD-L1 expression are shown in Table 3.

## 4. Ipilimumab—Cytotoxic T-Lymphocyte-Associated Protein 4 (CD152) Blocker

Ipilimumab, a monoclonal antibody developed by Bristol-Myers Squibb, represents a landmark advancement in cancer immunotherapy. In 2011, it became the first immune checkpoint inhibitor to receive U.S. Food and Drug Administration (FDA) approval—initially for the treatment of metastatic melanoma—marking a pivotal shift in oncology. This approval established immunotherapy as a viable treatment option for malignancies unresponsive to conventional approaches such as chemotherapy and radiation [178,288,289].

The therapeutic rationale for ipilimumab centers on its selective inhibition of cytotoxic T-lymphocyte-associated protein 4 (CTLA-4), a critical immune checkpoint that downregulates T-cell activation to maintain immune homeostasis and prevent autoimmunity. Tumors frequently exploit this pathway to evade immune surveillance. CTLA-4, expressed on activated T cells, competes with the co-stimulatory receptor CD28 for binding to B7-1 (CD80) and B7-2 (CD86) on antigen-presenting cells. By blocking CTLA-4, ipilimumab lifts this inhibitory signal, thereby promoting T-cell activation, proliferation, and the generation of a durable antitumor immune response [289,290].

The clinical success of ipilimumab in metastatic melanoma solidified the therapeutic potential of immune checkpoint blockade and positioned it as a cornerstone in the evolution of modern immuno-oncology [288,289,290].

Immune-modulating receptor–related drug targets are summarized in Table 4.

Combining ICIs—particularly CTLA-4 and PD-1 blockers such as ipilimumab and nivolumab—has shown synergistic antitumor activity in numerous clinical studies [291,292,293], leading to improved outcomes across a range of malignancies [294,295,296]. Additional trials further support this benefit [297,298,299], with subsequent studies confirming efficacy across tumor types [300,301,302] and highlighting long-term outcomes [303,304,305,306]. Ipilimumab is now approved by the U.S. Food and Drug Administration (FDA) for multiple cancer types, including melanoma, non-small cell lung cancer (NSCLC), renal cell carcinoma (RCC), genitourinary malignancies, and hepatocellular carcinoma [307,308,309], with further approvals documented in later studies [310,311,312]. In melanoma, landmark trials have demonstrated significant improvements in OS and PFS [313,314,315], firmly establishing ipilimumab as a mainstay therapy [316,317]. The combination of ipilimumab with nivolumab further enhances clinical benefit by increasing response rates and survival across tumor types [168,318]. Beyond melanoma, ipilimumab has been evaluated in NSCLC, RCC, and castration-resistant prostate cancer (CRPC) [319,320,321], often in combination with chemotherapy [322,323,324], underscoring its broad immunomodulatory potential [325,326,327].

Emerging evidence suggests a potential role for ipilimumab in oral cancers, particularly OSCC—a malignancy characterized by aggressive clinical behavior, high recurrence rates, and frequent late-stage presentation. Like other solid tumors, OSCC employs immune evasion strategies, including CTLA-4 upregulation [328]. Targeting CTLA-4 may therefore represent a novel therapeutic strategy for advanced or treatment-refractory OSCC [329,330,331,332]. Early-phase studies in HNSCC—which encompasses OSCC—have shown that ipilimumab, either alone or combined with chemotherapy and radiation, can induce immune activation and, in certain cases, tumor regression [333,334,335,336,337]. Notably, neoadjuvant checkpoint blockade with nivolumab, alone or with a single dose of ipilimumab prior to surgery, has been reported as feasible and potentially active in HNSCC [338,339,340,341].

The therapeutic potential of ipilimumab in OSCC is particularly evident when combined with PD-1 inhibitors such as pembrolizumab or nivolumab. These regimens may overcome immune resistance in patients who do not respond to conventional therapy, offering the possibility of durable tumor control [342,343]. Some clinical reports have documented sustained responses in OSCC patients receiving ipilimumab-based regimens, highlighting its potential utility in high-risk disease [37,344].

Despite these promising findings, several challenges complicate the integration of ipilimumab into OSCC treatment. Variable CTLA-4 expression and ligand heterogeneity limit efficacy to certain patient subgroups [345,346,347], and reliable predictive biomarkers for response are lacking [348,349,350]. Furthermore, ipilimumab is associated with a relatively high incidence of irAEs affecting the skin, gastrointestinal tract, liver, endocrine organs, and cardiovascular system, necessitating careful monitoring [351,352,353], with additional studies detailing incidence and management strategies [354,355,356,357,358,359]. Most irAEs are manageable with immunosuppressive therapy, and early detection through proactive monitoring can improve patient outcomes [293,360].

Combination strategies may enhance therapeutic benefit in OSCC. Radiotherapy, for instance, can increase tumor immunogenicity and upregulate immune checkpoint targets, potentially augmenting ipilimumab efficacy in locally advanced or recurrent disease [37,361]. Similarly, ipilimumab–chemotherapy combinations have produced higher response rates in select studies [362,363]. Neoadjuvant use of ipilimumab—administered before surgical resection—has shown promise in improving resectability and reducing recurrence risk in OSCC, with early trials reporting improved tumor control and favorable surgical outcomes [339,364]. Ongoing research is also investigating multi-agent immunotherapy and targeted therapy combinations to address resistance mechanisms and optimize outcomes [365,366].

Ipilimumab’s CTLA-4 blockade has been transformative in the evolution of cancer immunotherapy, reshaping treatment paradigms for difficult-to-treat malignancies. Although its role in OSCC remains under active investigation, preliminary data are encouraging. Advances in biomarker discovery and rational combination strategies may broaden its applicability and improve patient selection [367,368].

Nevertheless, notable limitations remain. Compared with PD-1/PD-L1 inhibitors, ipilimumab generally has a lower objective response rate and slower onset of action—potentially problematic in rapidly progressing disease [178,369]. Its higher toxicity burden can limit use in elderly patients or those with significant comorbidities [370]. While combination therapy improves efficacy, it also increases the likelihood of severe irAEs—particularly colitis, hepatitis, and hypophysitis [345,371,372,373]—as well as dermatitis, which often requires corticosteroid therapy or treatment discontinuation [374,375,376]. Additional reports highlight management strategies and outcomes in affected patients [377,378,379]. These adverse effects are typically more severe than those seen with PD-1 blockade. In addition, patients with pre-existing autoimmune diseases—often excluded from pivotal trials—represent a high-risk group with unpredictable safety outcomes [213,380]. The absence of validated biomarkers for predicting both efficacy and toxicity continues to hinder personalized therapy [381,382].

Management of irAEs, including oral mucosal toxicities such as xerostomia and candidiasis, requires a multidisciplinary approach. Coordination among dental specialists, nutritionists, and infectious disease experts is crucial to preserve oral health, prevent complications, and maintain nutritional status [383,384]. Dental interventions can mitigate mucosal injury, nutritional support can address swallowing and dietary limitations, and infectious disease management ensures prompt treatment of opportunistic infections, which are common in immunocompromised patients [385].

Personalized care protocols—including irAE severity grading, therapy modification, and standardized monitoring frameworks—are essential to optimize the risk–benefit profile of ipilimumab [386]. Early intervention protocols reduce the incidence of severe complications [387]. Incorporating biomarker-guided strategies, such as PD-L1 assessment or liquid biopsy monitoring of tumor and immune dynamics, may further enable precise and adaptive treatment approaches, improving both safety and efficacy in ipilimumab-based regimens [388].

**Figure 2:**
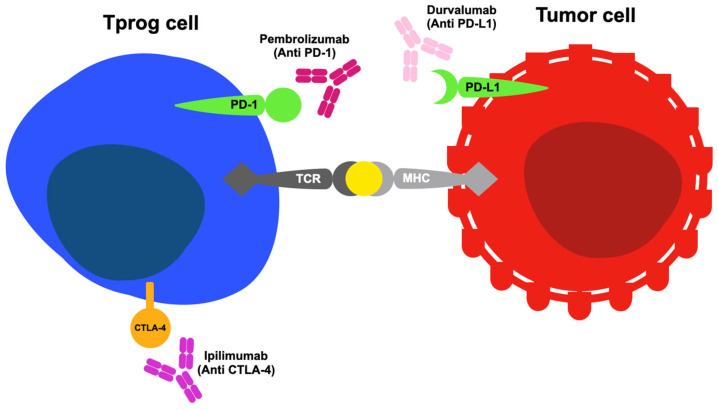
Mechanisms of action of selected immune checkpoint inhibitors, according to [389], including pembrolizumab, durvalumab, and ipilimumab, where Tprog cell—progenitor-like exhausted T cell. T-cell activation requires two separate signals: the first involves the T-cell receptor (TCR) recognizing antigen–MHC (major histocompatibility complex) complexes, while the second comes from the interaction of co-stimulatory molecules with their respective receptors on the T-cell surface. In the tumor microenvironment (TME), however, co-inhibitory signals become upregulated, hindering T-cell activation by engaging with their corresponding receptors (such as PD-1/CD279 with PD-L1/CD274, CTLA-4 with B7-1/CD80 and B7-2/CD86). Blocking the CTLA-4 and PD-1/PD-L1 pathways enhances immune responses in different ways. Inhibiting CTLA-4 boosts the activation and expansion of diverse T-cell populations while decreasing the suppressive effects of regulatory T cells. Blocking the PD-1 pathway reactivates dormant T cells that target tumors. Using both blockades together may produce a stronger and more sustained immune attack against cancer; pembrolizumab, durvalumab, and ipilimumab can partially reactivate T cells by blocking these inhibitory pathways.

Figure 2 illustrates the primary modes of action of selected ICIs.

## 5. Implications and Observations: Toward a Research Outlook

Although many pivotal immunotherapy trials have been conducted in the broader HNSCC population, recent subgroup analyses have yielded important insights for OSCC. In the KEYNOTE-048 study, pembrolizumab with or without chemotherapy improved survival in recurrent/metastatic HNSCC, and subgroup analyses confirmed consistent efficacy in oral cavity cancers, though benefit varied across subsites [119,390]. A phase II trial of perioperative pembrolizumab in locally advanced resectable HNSCC, which included a substantial proportion of OSCC patients, showed promising pathological response rates, underscoring the relevance of ICIs for this disease [391]. OSCC-focused studies have also tested neoadjuvant immunotherapy, where early biomarker-driven strategies are being explored [392,393]. Together, these findings highlight the importance of accounting for OSCC-specific biology and clinical outcomes in therapeutic development.

Because of the heterogeneity of head and neck cancers, we prioritized studies specifically addressing OSCC. Where available, oral cavity subgroup data from broader HNSCC trials are included to increase disease-specific relevance. For example, subgroup analyses from KEYNOTE-048 and perioperative pembrolizumab studies demonstrated consistent benefit in oral cavity cancers [119,391]. This ensures that therapeutic interpretations and biomarker considerations remain directly applicable to OSCC, rather than extrapolated from mixed HNSCC cohorts [394].

Recent phase III trial readouts (2022–2025) have significantly expanded the evidence base. The 4–5-year analyses of KEYNOTE-048 confirmed the durable survival benefit of pembrolizumab (alone or with chemotherapy) versus the EXTREME regimen, with the greatest advantage in PD-L1 CPS ≥ 20 subgroups [119,177]. In contrast, durvalumab with or without tremelimumab in KESTREL and EAGLE did not improve OS, though toxicity was lower and exploratory analyses suggested possible benefit in high-TMB subsets [33,215]. Similarly, CheckMate-651 with nivolumab plus ipilimumab did not meet its OS endpoint but showed a favorable toxicity profile compared to chemotherapy [395]. More recently, a pivotal phase III perioperative pembrolizumab trial demonstrated significant improvements in event-free survival among patients with resectable HNSCC, including OSCC, with favorable overall survival trends [391]. Collectively, these results refine the therapeutic positioning of pembrolizumab, durvalumab, and ipilimumab in OSCC.

While PD-L1 expression and TMB are established biomarkers for checkpoint blockade, their predictive value in OSCC remains inconsistent [396]. Emerging evidence suggests the gut and oral microbiome may influence ICI responses, with enrichment of *Akkermansia muciniphila* and Faecalibacterium prausnitzii associated with improved survival in epithelial tumors [51,52,397]. OSCC-specific studies also suggest that salivary and oral microbiota composition affects therapeutic outcomes [392,393]. Additional candidates, including DNA methylation, histone modifications, and immune gene expression profiles, are being investigated [397,398], but validation across cohorts and standardization of assays remain major challenges [399].

Checkpoint inhibitor activity in OSCC is modulated by multiple biomarkers and host factors. PD-L1 CPS is the only validated predictive biomarker, though variability in scoring and intratumoral heterogeneity limit accuracy [119,400]. Tumor mutational burden and interferon-γ-related gene signatures show promise but remain technically challenging due to assay variability [33,395]. Increasingly, the gut and oral microbiomes have been implicated, with taxa such as *Akkermansia* and *Faecalibacterium* linked to favorable responses [52,401,402]. OSCC-specific studies suggest salivary and oral microbiome diversity may correlate with neoadjuvant responses [403,404], though results vary due to sampling site, sequencing platform, and confounding variables such as diet, antibiotics, or proton pump inhibitors [405]. Resistance arises through intrinsic mechanisms—including antigen presentation defects, JAK1/2 alterations, and oncogenic PI3K/AKT and WNT/β-catenin signaling—and extrinsic mechanisms such as Tregs, myeloid suppressors, CAF-driven stromal exclusion, and hypoxia [277,406]. These findings underscore the need for integrated biomarker strategies and combinatorial approaches.

Defects in antigen presentation are a key tumor-intrinsic resistance mechanism in OSCC. Downregulation or loss of MHC class I molecules—caused by genetic, epigenetic, or post-transcriptional alterations in β_2_-microglobulin, TAP, and NLRC5—reduces immunogenicity and often correlates with poor prognosis. Importantly, such defects may be reversible, offering therapeutic opportunities to restore antigen presentation [407]. Some OSCC tumors also exploit non-classical MHC molecules (HLA-G, HLA-E) or downregulate co-stimulatory molecules such as CD80/CD86, further impairing T-cell activation [408].

The TME strongly influences immunotherapy response. Immunologically “cold” tumors with low immune infiltration and dominant immunosuppressive signaling often resist checkpoint blockade [51,409]. Approaches to convert cold tumors into inflamed, responsive phenotypes include radiotherapy-induced antigen release, VEGF or TGF-β blockade, oncolytic viruses, STING agonists, and microbiome modulation [52,399]. The TME of OSCC is highly immunosuppressive, characterized by Tregs, MDSCs, and TAMs producing IL-10 and TGF-β, hypoxia driven by HIF signaling with lactic acid accumulation, and CAF-mediated stromal exclusion [410,411,412,413,414]. Dysbiosis of the oral microbiota, such as enrichment of *Porphyromonas gingivalis* or *Fusobacterium nucleatum*, further promotes inflammation, oncogenic signaling, and immune evasion [410].

To counter this immune escape, several novel therapeutic modalities are under investigation. Beyond PD-1/PD-L1 and CTLA-4, inhibitory pathways such as LAG-3, TIM-3, TIGIT, and VISTA are promising targets [415]. Combination strategies—including immunotherapy with radiotherapy, chemotherapy, targeted agents, or anti-angiogenic therapy—may enhance immunogenicity and overcome resistance. Anti-angiogenic drugs can normalize vasculature and improve immune cell infiltration [416]. Adoptive T-cell transfer and CAR T-cell therapy targeting OSCC-specific antigens (e.g., EGFR, MUC1) have shown encouraging preclinical results [417], while oncolytic viruses and cancer vaccines may synergize with checkpoint inhibitors and cellular therapies [418]. LAG-3, TIM-3, TIGIT, and VISTA inhibitors are being tested in early-phase trials, with preliminary evidence of synergy with PD-1/PD-L1 blockade [419,420,421,422].

Clinically, pembrolizumab plus platinum/5-FU is now established as a first-line standard in recurrent/metastatic OSCC, particularly in CPS ≥ 1 disease [119,177]. In the perioperative setting, pembrolizumab has shown superiority over surgery with adjuvant therapy alone, establishing immunotherapy as a promising new standard in resectable OSCC [391]. By contrast, concurrent PD-(L)1 blockade with definitive chemoradiation has not improved outcomes, underscoring the importance of sequencing [423]. Cetuximab-based regimens remain an option post–PD-1 therapy [424], whereas dual PD-1/CTLA-4 blockade has not demonstrated OS benefit in phase III trials [395]. Combination approaches, while potentially more effective, increase irAEs and require vigilant monitoring, early intervention, and careful patient selection. Sequential rather than concurrent administration may reduce toxicity while preserving efficacy [30,289].

Acquired resistance to ICIs remains a major obstacle. Mechanisms include mutations in B2M or HLA genes, impaired interferon-γ signaling via JAK1/2 mutations, and adaptive upregulation of checkpoints such as TIM-3, LAG-3, and TIGIT [51,409]. Strategies to address this include bispecific antibodies, checkpoint combinations, personalized neoantigen vaccines, and adoptive T-cell therapies, all aiming to restore immune recognition and sustain durable responses.

Combination therapies remain central to OSCC immunotherapy. Chemotherapy, radiotherapy, and targeted agents can augment immunogenicity, while dual checkpoint blockade with PD-1 plus CTLA-4, LAG-3, or TIGIT inhibitors shows early promise but increases toxicity. Adoptive cell therapies, including TIL and CAR T-cell therapy, offer additional avenues, though efficacy is limited by the immunosuppressive TME.

Integration of tumor-intrinsic features, the TME, and emerging biomarkers is critical for personalized strategies. Beyond PD-L1 and TMB, microbiome signatures, epigenetic modifications, and transcriptional profiles may guide patient selection, therapeutic monitoring, and adaptive combinations to overcome resistance. Overall, the evolving immunotherapy landscape in OSCC underscores the importance of mechanistic understanding, rational combination strategies, and biomarker-driven personalization to achieve durable benefit.

## 6. Conclusions

The advent of ICIs—including pembrolizumab, durvalumab, and ipilimumab—has significantly transformed the therapeutic landscape of OSCC. These agents have demonstrated notable OS and PFS, particularly in recurrent or metastatic disease where conventional modalities such as surgery, chemotherapy, and radiation offer limited benefit. Despite these advances, the clinical application of ICIs is often constrained by the high incidence of irAEs, necessitating vigilant, personalized management strategies to mitigate toxicity without compromising therapeutic efficacy.

A critical limitation in the current evidence base is the paucity of OSCC-specific data. Most clinical trials evaluating ICIs have been conducted within broader HNSCC populations, which may not adequately reflect the distinct biological behavior and clinical course of OSCC. This underscores the urgent need for dedicated OSCC-focused studies and the identification of reliable predictive biomarkers to inform treatment selection and optimize outcomes.

Importantly, recent findings indicate that the magnitude of survival benefit from ICIs varies significantly between monotherapy and combination regimens. This variability reinforces the need for individualized treatment planning that carefully balances efficacy with tolerability. As combination therapies often carry a higher risk of irAEs, evolving management strategies—particularly those targeting oral toxicities—are essential for maintaining patient quality of life while preserving antitumor immune responses.

Emerging research also points to the interplay between the host microbiome and PD-L1 expression as a potential predictive biomarker of ICI responsiveness. This relationship opens new avenues for integrating microbiome modulation into immunotherapeutic strategies, with the potential to enhance treatment efficacy and overcome resistance.

In parallel, ongoing investigations into the synergistic potential of ICIs combined with targeted agents, chemoradiotherapy, and novel immunotherapies—such as chimeric antigen receptor (CAR) T-cell therapy and natural killer (NK) cell-based approaches—are expanding the therapeutic armamentarium. These multimodal strategies aim to circumvent resistance mechanisms inherent to ICI monotherapy and extend durable responses to a broader patient population.

Personalized medicine, guided by comprehensive genomic, transcriptomic, and immune profiling, is increasingly recognized as central to optimizing treatment for OSCC. Precision-based approaches can refine therapeutic selection and dosing, minimize adverse effects, and enhance the durability of response by tailoring interventions to the unique molecular and immunological characteristics of each tumor.

While ICIs represent a significant advancement in OSCC management, key challenges remain. These include improving treatment specificity, managing irAEs—particularly oral mucosal toxicities such as xerostomia and mucositis—identifying robust predictive biomarkers, and overcoming primary and acquired resistance. Addressing these challenges through continued research and innovation is essential for fully realizing the promise of immunotherapy in OSCC.

Future directions should prioritize the development of dynamic, toxicity-guided management protocols. For example, adjusting immunotherapy dosing in response to the severity of salivary gland dysfunction could help preserve quality of life while maintaining efficacy. Moreover, deeper exploration of the microbiome–PD-L1 axis may yield novel predictive models that facilitate more personalized, effective treatment strategies. Collectively, these advancements will be critical in advancing ICI therapy and improving long-term outcomes for patients with OSCC.

## Figures and Tables

**Table 1:** Selected ICIs in cancer immunotherapy according to [46,47,48].

Checkpoint Inhibitor (Approval Year); Checkpoint Target; Ig Type	Mechanism of Action	Conditions	Investigational	Treatment-Related Side Effects
Pembrolizumab (Keytruda, 2014); PD-1; IgG4	Binds PD-1 on activated T cells, blocking its interaction with PD-L1/PD-L2. This prevents inhibitory signaling, restores T-cell activation, and enhances antitumor immunity while minimizing Fc-mediated cytotoxicity.	Head and neck squamous cell carcinoma (HNSCC), Recurrent, locally advanced, or metastatic esophageal squamous cell carcinoma (ESCC), Hepatocellular carcinoma (HCC), Merkel cell carcinoma (MCC), Malignant pleural mesothelioma (MPM), Non-small cell lung cancer (NSCLC), Metastatic small cell lung cancer (SCLC), Recurrent cutaneous squamous cell carcinoma (cSCC), Renal cell carcinoma (RCC), Urothelial carcinoma (UC), Triple-negative breast cancer (TNBC), Microsatellite instability-high (MSI-H) or Mismatch repair (MMR)-deficient solid tumors, Gastroesophageal junction (GEJ) cancer, Cervical cancer, Endometrial cancer, Stomach cancer, Classical Hodgkin’s lymphoma, Metastatic melanoma	Renal transitional cell carcinoma (TCC), MSI-H/dMMR (deficient DNA mismatch repair) noncolorectal cancer, Brain tumor, Metastatic HER2-negative breast cancer, Metastatic anal cancer, Lymphoma, Pancreatic cancer, Recurrent glioblastoma, Refractory esophageal cancer	Fatigue, Skin adverse reactions, Arthralgia, Pneumonitis, Colitis Hepatitis, Endocrinopathies, Nephritis
Durvalumab (Imfinzi, 2017); PD-L1; IgG1;	Binds PD-L1, blocking its interaction with PD-1 and CD80, thereby preventing T-cell inhibition and promoting immune-mediated tumor destruction	HCC, NSCL, SCLC, Metastatic UC	Extensive-stage small-cell lung cancer (ES-SCLC), Recurrent and/or metastatic HNSCC Non-muscle invasive bladder cancer, Anal cancer, Breast neoplasms, Cervical cancer, Colorectal neoplasms Lymphoma, Mesothelioma, Solid tumors, Esophageal cancer, Nasopharyngeal carcinoma	Hepatitis, Pneumonitis, Colitis, Endocrinopathies
Ipilimumab (Yervoy, 2011); CTLA-4; IgG1;	Binds CTLA-4 on activated T cells, preventing its interaction with B7-1 (CD80) and B7-2 (CD86) on antigen-presenting cells. This blocks inhibitory signaling, promotes co-stimulatory CD28 signaling, and increases T-cell proliferation and antitumor activity.	MPM, NSCLC, Advanced RCC, Metastatic melanoma, dMMR colorectal cancer, HCC—in combination with Nivolumab, ESCC—in combination with Nivolumab	SCLC, Advanced UC Prostate cancer, Solid tumors, Untreated and advanced melanoma	Colitis, Hepatitis, Dermatitis, Neuropathies, Endocrinopathies, Pneumonitis, Nephritis, Encephalitis

**Table 2:** Effector immune responses triggered by PD-1 blockade according to [129].

Immunological Action
Dendritic cells
Elevated expression of CD40/CD40L, promoting dendritic cell (DC) survival and apoptosis resistance; Prolonged DC lifespan; Recruitment and activation of T cells, stimulating local antitumor immune responses; Enhanced IL-12 secretion in response to T cells activated by anti-PD-1 therapy; Facilitating cross-talk between adaptive and innate immunity, enabling tumor-specific immune responses.
Macrophages/monocytes
Enhanced infiltration of tumor-associated macrophages (TAMs); Elevated M1-to-M2 macrophage ratio, linked to better prognosis and decreased tumor burden; Augmented phagocytic activity against tumor cells; Upregulation of IL-12 secretion and activation of signal transducer and activators of transcription 1 (STAT1) cellular messaging; Increased production of IL-6.
Natural killer cells
Enhanced tumor infiltration; Restoration of cytotoxic activity following immune suppression in the tumor microenvironment; Elevated cell proliferation and differentiation; Increased secretion of granzyme B, perforin, and interferon gamma (IFN-γ).
T cells
Amplified expansion of specific T-cell populations; Upregulated production of effector cytokines by infiltrating T cells; Heightened levels of IFN-γ and tumor necrosis factor alpha (TNF-α) expression; Greater infiltration of T cells into tumor tissue; Antigen-specific immune activation driven by T cells.

**Table 3:** Regulation of PD-L1 by tumor microenvironment (TME) according to [287], where p65 refers to the subunit of the transcription factor NF-κB, NRF1—nuclear respiratory factor 1, miR-15b-5p—microRNA-15b-5p, STAT3—signal transducer and activator of transcription 3, IL-6—interleukin-6, NF-κB—nuclear factor-kappa B, Smad2/3—Sma and Mad intracellular signaling proteins family member 2/3, IFN-γ—interferon-gamma, HK2—hexokinase 2, IκBα T291—IkappaB alpha amino acid residue number 291, miRNA—microRNA, mRNA—messenger RNA, NSCLC—non-small cell lung cancer, M2—anti-inflammatory macrophages, CD8—cluster of differentiation 8.

Regulatory Factor	Mechanism/Pathway	Effect on PD-L1 Expression	Cancer Type	Therapeutic Implications
IL-17A	Activates p65/NRF1/miR-15b-5p axis	Increases PD-L1 expression	Colorectal cancer	Promotes resistance to anti-PD-1 therapy
IL-6	Activates the STAT3 pathway	Induces PD-L1 on bone marrow cells	Glioblastoma	Blocking IL-6 inhibits tumor growth, improves survival
TNF-α	Activates the NF-κB pathway	Induces PD-L1 on mast cells	Gastric cancer	Promotes immune evasion and tumor progression
TGF-β (from TAMs)	Inhibits Smad2/3 phosphorylation and mitochondrial respiration	Suppresses T-cell activity, indirectly sustaining PD-L1 effects	General tumor TME	Reduces IFN-γ and granzyme B production
High Glucose	Causes HK2 dissociation, IκBα T291 phosphorylation, NF-κB activation	Upregulates PD-L1 transcriptionally	Glioblastoma	HK2 inhibition + anti-PD-1 synergistically reduces tumor burden
Exosomes (general)	Transports miRNAs, mRNAs, proteins	Mediates PD-L1 regulation via intercellular signaling	Various cancers	Key regulators of immune escape
PD-L1 Splice Variants (Exosomal)	Lacks transmembrane domain; acts as decoys	Induces resistance to PD-L1 blockade	NSCLC (aPD-L1-resistant)	Limits the effectiveness of anti-PD-L1 antibodies
Glioblastoma Stem Cell Exosomes	Activates STAT3, M2 macrophage polarization	Enhances PD-L1 expression on macrophages	Glioblastoma	Promotes immune suppression in TME
Metastatic Melanoma Exosomes	Carries PD-L1 on surface, responsive to IFN-γ	Increases circulating PD-L1 levels	Melanoma	Correlates with suppressed CD8 T-cell activity and tumor growth

**Table 4:** Chosen drug targets involving immune-modulating receptors according to [126], where AP-1—activator protein 1; CD—cluster of differentiation; CTLA-4—cytotoxic T-lymphocyte-associated protein 4; Eomes—eomesodermin; ERK—extracellular signal-regulated kinase; GATA3—GATA binding protein 3; HLA—human leukocyte antigen; ICOS—inducible T-cell co-stimulator; JNK, Janus kinase; MAPK—mitogen-activated protein kinase; mTOR—mechanistic target of rapamycin; NFAT—nuclear factor of activated T cells; NF-κB—nuclear factor kappa-light-chain-enhancer of activated B cells; PD-1—programmed cell death protein 1; PI3K-AKT—phosphoinositide 3-kinase/protein kinase B; T-bet—T-box transcription factor expressed in T cells.

Inhibitory Receptors	Suppressed Effector Signaling Activity	Stimulatory Receptors	Enhanced Effector Signaling Activity
CTLA4 (CD152)	PI3K-AKT, AP-1, NF-κB, NFAT, MAPK	CD28	PI3K-AKT, ERK, T-bet, Eomes, GATA3, AP-1, NFAT, NF-κBdata
PD-1 (CD279)	MAPK, PI3K-AKT, AP-1, NFAT, NF-κBdata	ICOS	JNK, PI3K-AKT-mTOR-NFAT

## Data Availability

Data sharing is not applicable to this article.

## Abbreviations

The following abbreviations are used in this manuscript:

AP-1	Activator Protein 1
CD	Cluster of Differentiation
CD8	Cluster of Differentiation 8
cSCC	Cutaneous Squamous Cell Carcinoma (Recurrent)
CTLA-4	Cytotoxic T-Lymphocyte-Associated Protein 4
dMMR	Mismatch Repair-Deficient
Eomes	Eomesodermin
ERK	Extracellular Signal-Regulated Kinase
ESCC	Esophageal Squamous Cell Carcinoma (Recurrent, Locally Advanced, or Metastatic)
ATA3	GATA Binding Protein 3
GEJ	Gastroesophageal Junction
HCC	Hepatocellular Carcinoma
HLA	Human Leukocyte Antigen
HK2	Hexokinase 2
HNSCC	Head and Neck Squamous Cell Carcinoma
ICOS	Inducible T-Cell Co-Stimulator
IFN-γ	Interferon-Gamma
IL-6	Interleukin-6
irAEs	Immune-Related Adverse Events
IκBα T291	IkappaB Alpha (Amino Acid Residue 291)
JNK	Janus Kinase
M2	Anti-Inflammatory Macrophages
MAPK	Mitogen-Activated Protein Kinase
MCC	Merkel Cell Carcinoma
miR-15b-5p	MicroRNA-15b-5p
miRNA	MicroRNA
mRNA	Messenger RNA
MPM	Malignant Pleural Mesothelioma
MSI-H	Microsatellite Instability-High
MSI-H/dMMR	Microsatellite Instability-High/Deficient DNA Mismatch Repair (Non-Colorectal Cancer)
mTOR	Mechanistic Target of Rapamycin
NFAT	Nuclear Factor of Activated T Cells
NF-κB	Nuclear Factor Kappa-Light-Chain-Enhancer of Activated B Cells
NRF1	Nuclear Respiratory Factor 1
NSCLC	Non-Small Cell Lung Cancer
p65	Subunit of the Transcription Factor NF-κB
PD-1	Programmed Cell Death Protein 1
PD-L1	Programmed Death-Ligand 1
PI3K-AKT	Phosphoinositide 3-Kinase/Protein Kinase B
PI3K/Akt/mTOR	Phosphoinositide 3-Kinase/Protein Kinase B/Mechanistic Target of Rapamycin
RCC	Renal Cell Carcinoma
SCLC	Small Cell Lung Cancer (Metastatic)
Smad2/3	Sma and Mad Intracellular Signaling Proteins Family Member 2/3
STAT3	Signal Transducer and Activator of Transcription 3
T-bet	T-Box Transcription Factor Expressed in T Cells
TCC	Transitional Cell Carcinoma (Renal)
TNBC	Triple-Negative Breast Cancer
UC	Urothelial Carcinoma
Wnt/β-catenin	Wingless/Integrated and Beta-Catenin Signaling Pathway
